# DNA methylation in peripheral blood is associated with renal aging and renal function decline: a national community study

**DOI:** 10.1186/s13148-024-01694-y

**Published:** 2024-06-15

**Authors:** Po-Lung Yang, Tai-Shuan Lai, Yu-Hsiang Chou, Liang-Chuan Lai, Shuei-Liong Lin, Yung-Ming Chen

**Affiliations:** 1 Department of Geriatrics and Gerontology, National Taiwan University Hospital College of Medicine, National Taiwan University, Taipei, Taiwan; 2 Renal Division, Department of Internal Medicine, National Taiwan University, College of Medicine, No. 1, Jen-Ai Road, Section 1, Taipei, 100 Taiwan; 3 Institute of Epidemiology and Preventive Medicine, College of Public Health, National Taiwan University, Taipei, Taiwan; 4 Graduate Institute of Physiology, College of Medicine, National Taiwan University, Taipei, Taiwan; 5 Bioinformatics and Biostatistics Core, Center of Genomic and Precision Medicine, National Taiwan University, Taipei, Taiwan; 6 Research Center for Developmental Biology and Regenerative Medicine, National Taiwan University, Taipei, Taiwan; 7 Department of Internal Medicine, National Taiwan University Hospital, Bei-Hu Branch, Taipei, Taiwan

**Keywords:** DNA methylation, Renal aging, Estimated glomerular filtration rate, Chronic kidney disease, Peripheral blood mononuclear cell

## Abstract

**Background:**

Older patients are at risk for acute kidney injury and chronic kidney disease. Age-related increases in DNA methylation at CpG islands have been linked to aging-related diseases like cancer and cardiovascular disease, but the exact causal relationship between methylation in renal aging and other kidney diseases remains unclear. This study aimed to elucidate the methylation status of peripheral blood mononuclear cells (PBMCs) in the Asian population. Using human whole blood DNA methylation analysis from the Taiwan Biobank, we included participants with both whole blood genome-wide methylation data and follow-up data on serum creatinine. We investigated hyper- and hypomethylated genes in comparison of participants with higher and lower estimated glomerular filtration (eGFR) decline rate in overall cohort as well as in comparison of old and young participants in subgroup of participants with higher eGFR decline rate. Common genes and signaling pathways in both comparative analyses were identified.

**Results:**

Among 1587 participants in the analysis, 187 participants had higher eGFR decline rate. According to the comparison of methylation in participants with different eGFR declines and at different ages, respectively, we identified common hypermethylated genes, including *DNMT3A* and *GGACT*, as well as hypomethylated genes such as *ARL6IP5*, *CYB5D1*, *BCL6*, *RPRD2*, *ZNF451*, and *MIAT* in both participants with higher eGFR decline and those of older age. We observed associations between the methylation status of signaling pathways and aging as well as renal function decline. These pathways notably included autophagy, p38 mitogen-activated protein kinases, and sirtuins, which were associated with autophagy process and cytokine production.

**Conclusions:**

Through methylation analysis of PBMCs, we identified genes and signaling pathways which could play crucial roles in the interplay of renal aging and renal function decline. These findings contribute to the development of novel biomarkers for identifying at-risk groups and even for therapeutic agent discovery.

**Supplementary Information:**

The online version contains supplementary material available at 10.1186/s13148-024-01694-y.

## Background

The global rise in the elderly population highlights the growing prevalence of age-related health issues. Older people are more susceptible to both acute kidney injury (AKI) and chronic kidney disease (CKD) [1], largely due to age-related changes in the structure and function of the kidney. Factors such as renal mass, functional nephron count, and renal blood flow decrease with age, increasing the risk of AKI, which is a significant contributor to end-stage kidney disease (ESKD) in elderly CKD patients [1, 2]. Studies on animal models further demonstrated that cellular senescence and vascular rarefaction exacerbate kidney fibrosis in aged mice, indicating a close relationship between renal aging and disease progression [3]. Epigenetic modulations, particularly DNA methylation, play a crucial role in downregulating gene expression [4]. Age-related increases in DNA methylation at CpG islands have been linked to aging-related diseases like cancer and cardiovascular disease [5]. In the context of renal aging, increased DNA methylation has been observed in the promoters of genes such as *KLOTHO* and *NRF2*, both of which help mitigate renal fibrosis, thereby connecting biological methylation alterations to declining renal function [6, 7]. Changes in DNA methylation associated with age at the time of renal transplantation also serve as predictors of future kidney injuries [8]. However, obtaining renal tissue is challenging. Several studies have utilized whole blood DNA methylation data from humans and have shown that DNA methylation can predict age and is correlated with a lower estimated glomerular filtration rate (eGFR) and a higher risk of CKD [9]. Moreover, animal studies analyzing blood DNA have revealed dysregulation of methylation and demethylation enzymes in diabetic kidney disease, highlighting the potential of whole blood DNA methylation as a predictor of disease progression [10]. Additionally, our previous study demonstrated that ischemia–reperfusion injury-associated acute kidney injury (IRI-AKI) leads to hypermethylation in pericytes, contributing to the progression from AKI to CKD, indicating that methylation could be a therapeutic target [11].

Despite these findings, the exact causal relationship between methylation in renal aging and other kidney diseases remains unclear. There are several studies of methylation analysis of whole blood associated with CKD or its progression, but these studies were limited by factors such as small sample size, cross-sectional design, lack of replication, and the presence of comorbidities. Although a complementary study analyzing DNA methylation from kidney tissue of CKD patients and controls suggested epigenetic regulation of core fibrotic pathways in CKD [9], there is limited research on this topic within the Asian population.

Using human whole blood DNA methylation analysis from the Taiwan Biobank, we investigated common genes and signaling pathways in both aging and higher eGFR decline participants. This study aimed to elucidate the methylation status of peripheral blood mononuclear cells (PBMCs) in the Asian population and enhance our understanding of the role of methylation in renal aging and renal disease, uncovering potential genes and pathways for therapeutic development.

## Results

### Characteristics and methylation profile

**Fig. 1:**
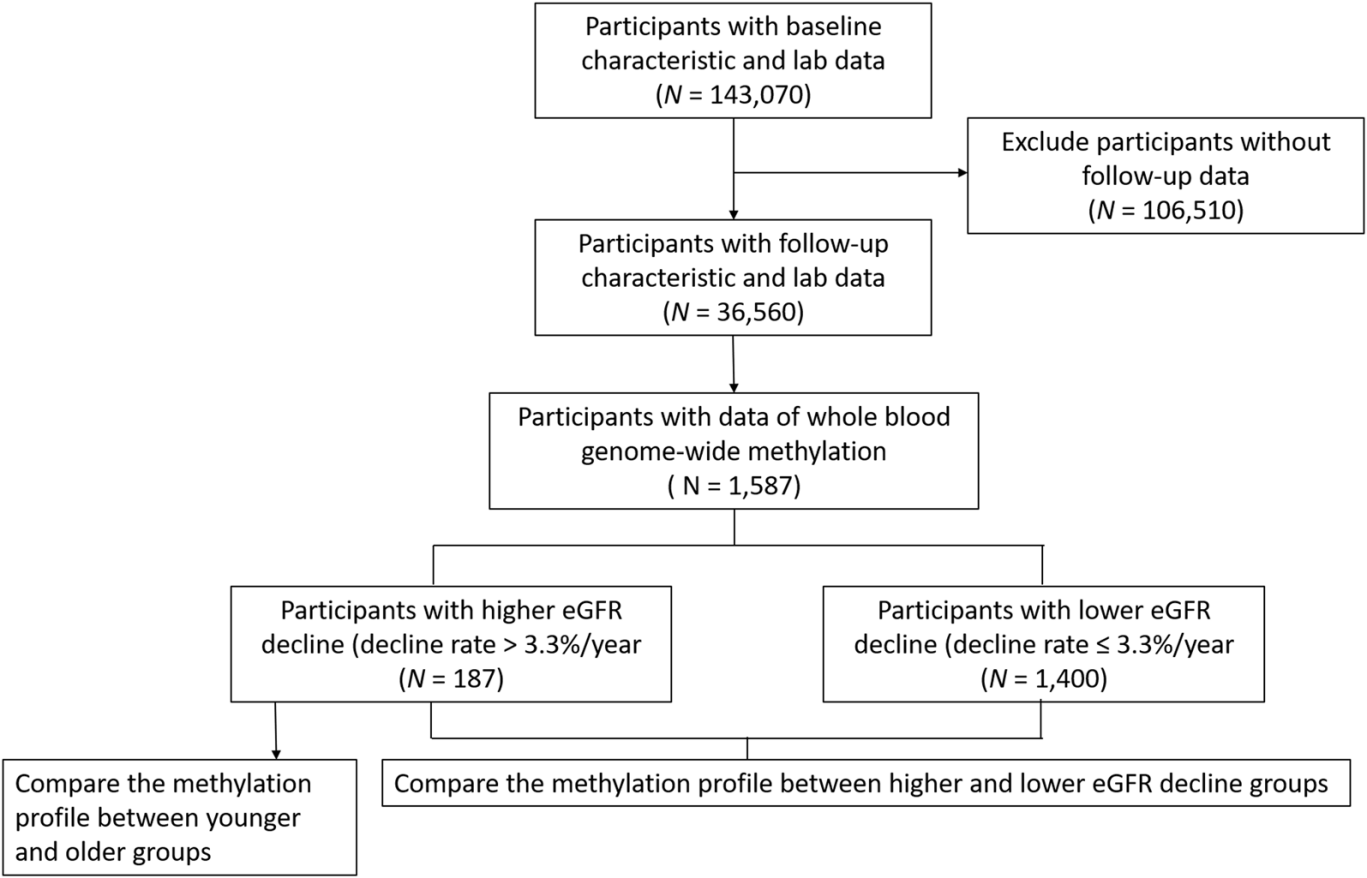
Flow diagram of participants enrollment

After screening 143,070 participants, we included 1587 participants with both whole blood genome-wide methylation data and follow-up data on serum creatinine. Subsequently, we categorized them into two groups based on whether their eGFR decline rate was more or less than 3.3% per year (Fig. 1). The baseline clinical characteristics of population with different eGFR decline rate are presented in Table 1. The baseline clinical characteristics of different age groups in the population with a higher eGFR decline rate are presented in Table 2. We observed that individuals with a higher rate of eGFR decline were significantly more likely to be male compared to those with a lower rate of eGFR decline. Additionally, those with a higher rate of eGFR decline had higher baseline body mass index (BMI), blood pressure, hemoglobin A1c, fasting glucose, triglyceride, and uric acid levels, but lower high-density lipoprotein, albumin, and eGFR. Within the subgroup of the population exhibiting a higher eGFR decline rate, we further identified that older individuals had lower baseline BMI, systolic blood pressure, eGFR and a higher eGFR decline rate.

**Table 1:** Baseline clinical characteristics of population with higher or lower eGFR decline rate

	All (*n* = 1,587)	eGFR decline rate > 3.3%/year (*n* = 187)	eGFR decline rate ≤ 3.3%/year (*n* = 1,400)	*P*
*Demographics*				
Age, years (mean, SD)	50.1 (10.8)	51.6 (10.3)	49.9 (10.9)	0.14
Sex, male, number (%)	789 (49.7)	111 (59.4)	678 (48.4)	0.01
BMI	24.33 (3.63)	25.14 (3.88)	24.22 (3.58)	< 0.01
Systolic blood pressure	116.51 (16.84)	121.66 (19.4)	115.82 (16.36)	< 0.01
Diastolic blood pressure	72.52 (11.05)	74.66 (11.27)	72.23 (10.99)	0.02
*Laboratory data (mean, SD)*				
WBC (1000/μl)	6 (1.52)	6.08 (1.45)	5.98 (1.52)	0.74
Platelet (1000/μl)	235.92 (54.61)	231.35 (55.86)	236.53 (54.44)	0.48
Hemoglobin (g/dl)	14 (1.56)	13.97 (1.61)	14.01 (1.56)	0.95
HbA1C (%)	5.72 (0.72)	5.91 (1.09)	5.69 (0.65)	< 0.01
Fasting glucose (mg/dl)	95.81 (18.33)	100.41 (25.19)	95.19 (17.12)	< 0.01
Total cholesterol (mg/dl)	195.47 (34.57)	193.32 (32.31)	195.76 (34.86)	0.66
Triglyceride (mg/dl)	118.36 (109.74)	144.86 (131.09)	114.82 (106.12)	< 0.01
HDL (mg/dl)	54.22 (13.76)	51.83 (14.24)	54.54 (13.67)	0.04
LDL (mg/dl)	121.99 (31.69)	117.73 (28.0)	122.56 (32.12)	0.15
Albumin (g/dl)	4.58 (0.26)	4.51 (0.39)	4.59 (0.23)	< 0.01
Total bilirubin (mg/dl)	0.7 (0.3)	0.7 (0.3)	0.7 (0.29)	0.99
AST (U/L)	24.36 (10.2)	25.7 (11.27)	24.18 (10.04)	0.16
ALT (U/L)	23.99 (19.34)	26.04 (18.71)	23.72 (19.42)	0.31
Uric acid (mg/dl)	5.61 (1.46)	5.97 (1.59)	5.57 (1.43)	< 0.01
UACR (mg/mg)	0.02 (0.05)	0.03 (0.03)	0.02 (0.05)	0.63
BUN (mg/dl)	13.23 (3.59)	13.52 (3.88)	13.2 (3.55)	0.51
1st Creatinine (mg/dl)	0.75 (0.19)	0.79 (0.19)	0.74 (0.19)	< 0.01
2nd Creatinine (mg/dl)	0.77 (0.21)	0.99 (0.28)	0.74 (0.18)	< 0.01
1st eGFR (ml/min/1.73 m^2^)	101.54 (13.99)	98.53 (14.34)	101.94 (13.9)	< 0.01
2nd eGFR (ml/min/1.73 m^2^)	97.21 (15.09)	80.01 (16.39)	99.5 (13.33)	< 0.01
eGFR decline rate (ml/min/1.73 m^2^/year)	1.01 (2.64)	5.49 (2.81)	0.41 (1.96)	0.02
eGFR decline rate (%/year)	1.01 (0.07)	5.48 (0.2)	0.41 (0.05)	< 0.01

*eGFR* estimated glomerular filtration rate, *SD* standard deviation, *BMI* body mass index, *WBC*  white blood cell, *HbA1C*  hemoglobin A1C, *HDL* high-density lipoprotein, *LDL*  low-density lipoprotein, *AST*  aspartate aminotransferase, *ALT*  alanine aminotransferase, *UACR* urine albumin-creatinine ratio, *BUN* blood urea nitrogen

**Table 2:** Baseline clinical characteristics of different age group with rapid renal function deterioration

	All (*n* = 187)	Group 1 (age < 45) (*n* = 46)	Group 2 (45 ≤ age < 65) (*n* = 123)	Group 3 (age ≥ 65) (*n* = 18)	*P*
*Demographics*					
Age, years (mean, SD)	51.58 (10.25)	37.5 (4.3)	54.57 (5.81)	67.11 (1.57)	< 0.01
Sex, male, number (%)	111 (59.35)	32 (69.57)	67 (54.47)	12 (66.67)	0.16
BMI	25.14 (3.88)	26.23 (5.23)	24.98 (3.32)	23.49 (2.53)	0.03
Systolic blood pressure	121.66(19.4)	111.9 (16.63)	123.61 (19.08)	133.25 (18.53)	< 0.01
Diastolic blood pressure	74.66 (11.27)	73.4 (12.05)	75.39 (11.23)	72.94 (9.49)	0.48
*Laboratory data (mean, SD)*					
WBC (1000/μl)	6 .08 (1.45)	6.21 (1.58)	6.07 (1.46)	5.75 (0.94)	0.52
Platelet (1000/μl)	231.35 (55.86)	238.8 (53.73)	232.03 (57.3)	207.61 (46.7)	0.13
Hemoglobin (g/dl)	13.97 (1.61)	14..29 (1.75)	13.83 (1.63)	14.08 (0.85)	0.25
HbA1C (%)	5.91 (1.09)	5.83 (1.32)	5.89 (0.99)	6.28 (1.07)	0.31
Fasting glucose (mg/dl)	100.41 (25.19)	96.3 (24.49)	100.84 (25.03)	108 (27.43)	0.24
Total cholesterol (mg/dl)	193.32 (32.31)	184.22 (29.12)	197.15 (33.19)	190.39 (30.50)	0.06
Triglyceride (mg/dl)	144.86 (131.09)	138.78 (98.36)	144.71 (114.1)	161.44 (261.91)	0.83
HDL (mg/dl)	51.83 (14.24)	47.59 (11.94)	53.11 (14.7)	53.94 (15.05)	0.06
LDL (mg/dl)	117.73 (28)	116.43 (26.84)	119.35 (28.34)	109.94 (28.59)	0.39
Albumin (g/dl)	4.51 (0.39)	4.58 (0.24)	4.49 (0.45)	4.48 (0.22)	0.37
Total bilirubin (mg/dl)	0.7 (0.3)	0.65 (0.29)	0.7 (0.3)	0.76 (0.35)	0.43
AST (U/L)	25.7 (11.27)	25.13 (12.59)	25.74 (11.01)	26.83 (9.92)	0.86
ALT (U/L)	26.04 (18.71)	29.93 (27.73)	24.6 (14.45)	25.89 (15.53)	0.26
Uric acid (mg/dl)	5.97 (1.59)	6.03 (1.62)	6 (1.64)	5.62 (1.16)	0.61
UACR (mg/mg)	0.03 (0.03)	0.01 (0.01)	0.03 (0.03)	0.05 (0.04)	0.16
BUN (mg/dl)	13.52 (3.88)	11.96 (2.81)	13.79 (4.07)	15.67 (3.63)	< 0.01
1st Creatinine (mg/dl)	0.79 (0.19)	0.78 (0.18)	0.79 (0.19)	0.83 (0.2)	0.60
2nd Creatinine (mg/dl)	0.99 (0.28)	0.94 (0.17)	0.99 (0.31)	1.11 (0.3)	0.10
1st eGFR (ml/min/1.73 m^2^)	98.53 (14.34)	111.32 (10.99)	95.63 (12.49)	85.69 (11.54)	< 0.01
2nd eGFR (ml/min/1.73 m^2^)	80.01 (16.39)	92.71 (11.98)	77.5 (14.7)	64.75 (16.71)	< 0.01
eGFR decline rate (ml/min/1.73 m^2^/year)	5.49 (2.81)	4.90 (1.80)	5.54 (3.01)	6.57 (3.32)	0.01
eGFR decline rate (%/year)	5.48 (0.21)	4.94 (0.27)	5.55 (0.27)	6.57 (0.78)	< 0.01

*eGFR* estimated glomerular filtration rate, *SD*  standard deviation, *BMI* body mass index, *WBC* white blood cell, *HbA1C* hemoglobin A1C, *HDL* high-density lipoprotein, *LDL* low-density lipoprotein, *AST*  aspartate aminotransferase, *ALT* alanine aminotransferase, *UACR* urine albumin-creatinine ratio, *BUN* blood urea nitrogen

### Identification of common genes in both groups with higher eGFR decline and aging

**Fig. 2:**
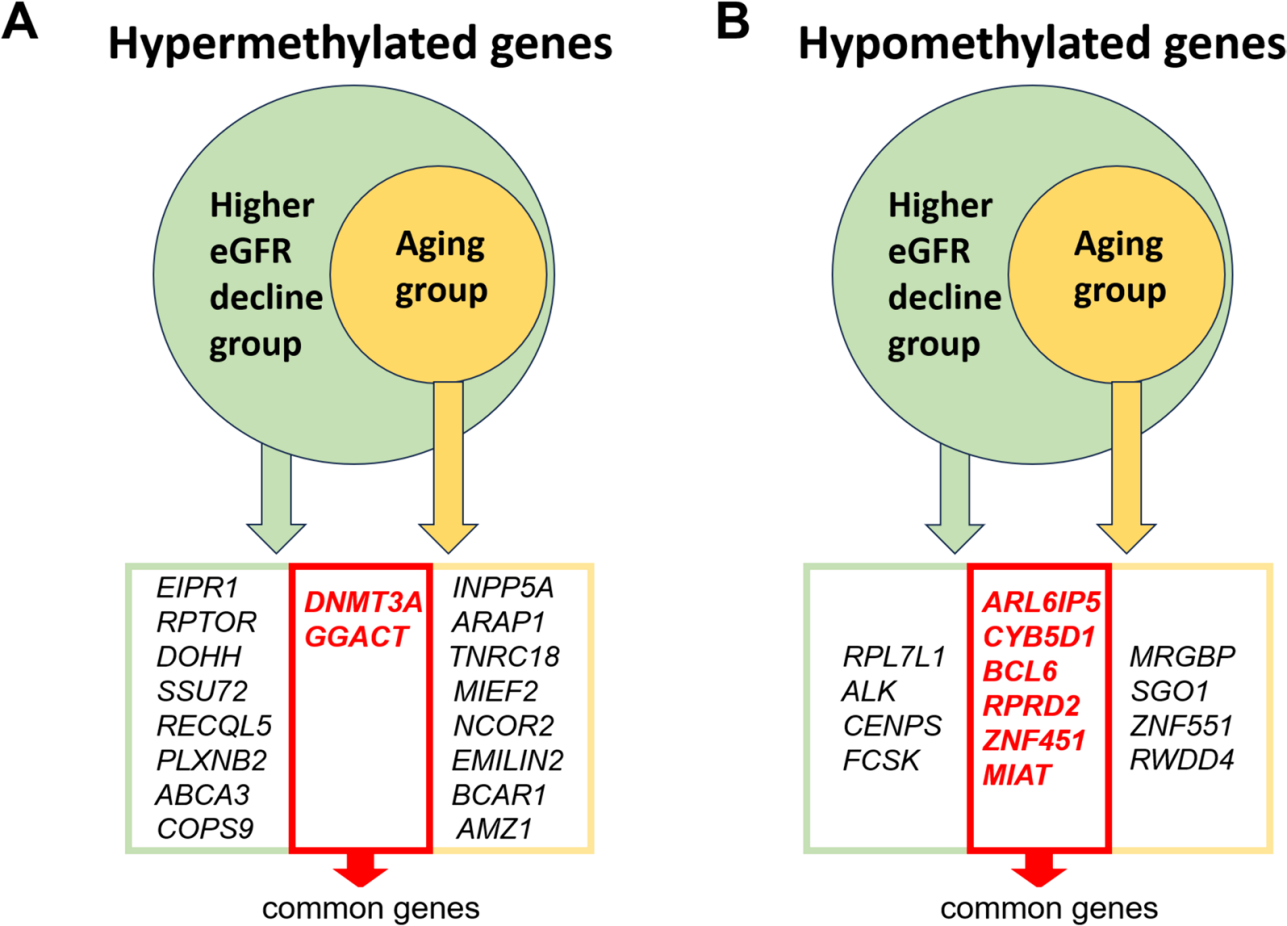
Identification of common **A** hypermethylated and **B** hypomethylated genes in comparison of groups with higher and lower eGFR decline rate and in comparison of old and young participants in subgroup of higher decline rate in eGFR

We initially compared the methylation status of genes between participants exhibiting a higher and lower eGFR decline rate (*n* = 1587). The most noteworthy hypermethylated genes in individuals with a higher eGFR decline rate comprised *EIPR1*, *RPTOR*, *DOHH*, *SSU72*, *DNMT3A*, *RECQL5*, *PLXNB2*, *ABCA3*, *GGACT*, and *COPS9* (Fig. 2A). In contrast, several genes displayed significant hypomethylation, including *ARL6IP5*, *CYB5D1*, *RPL7L1*, *ALK*, *CENPS*, *ZNF451*, *FCSK*, *BCL6*, *RPRD2*, and *MIAT* (Fig. 2B).

In subgroup of participants exhibiting a higher decline rate in eGFR (*n* = 187), we conducted a comparison of gene methylation status between older and younger participants. Among older individuals, the most significantly hypermethylated genes included *INPP5A*, *ARAP1*, *TNRC18*, *DNMT3A*, *MIEF2*, *GGACT*, *NCOR2*, *EMILIN2*, *BCAR1*, and *AMZ1* (Fig. 2A). Conversely, several hypomethylated genes were also identified, such as *MRGBP*, *ARL6IP5*, *CYB5D1*, *BCL6*, *RPRD2*, *ZNF451*, *MIAT*, *SGO1*, *ZNF551*, and *RWDD4* (Fig. 2B).

The common genes between participants exhibiting a higher eGFR decline rate and those of old age included *DNMT3A*, *GGACT* with hypermethylation, and *ARL6IP5*, *CYB5D1*, *BCL6*, *RPRD2*, *ZNF451*, and *MIAT* with hypomethylation (Fig. 2).

### Functional analysis of differential methylation detectable in both groups experiencing higher eGFR decline and aging

To evaluate the functions of the differential methylated genes, ingenuity pathway analysis (IPA) was applied. Different functional categories were observed when the genes displaying differential methylation. In overall cohort (*n* = 1587), hypermethylated signaling pathways in participants with a higher eGFR decline rate were found to be involved in neuron signaling transduction pathway such as myelination signaling pathway, synaptic long-term potentiation, and netrin signaling (Additional file 1: Fig. S2A). In contrast, hypomethylated signaling pathways in participants with a higher eGFR decline rate were found to be involved in different basal cellular process, including kinetochore metaphase signaling pathway, insulin receptor signaling, autophagy, and protein kinase a signaling (Additional file 1: Fig. S2B).

**Fig. 3:**
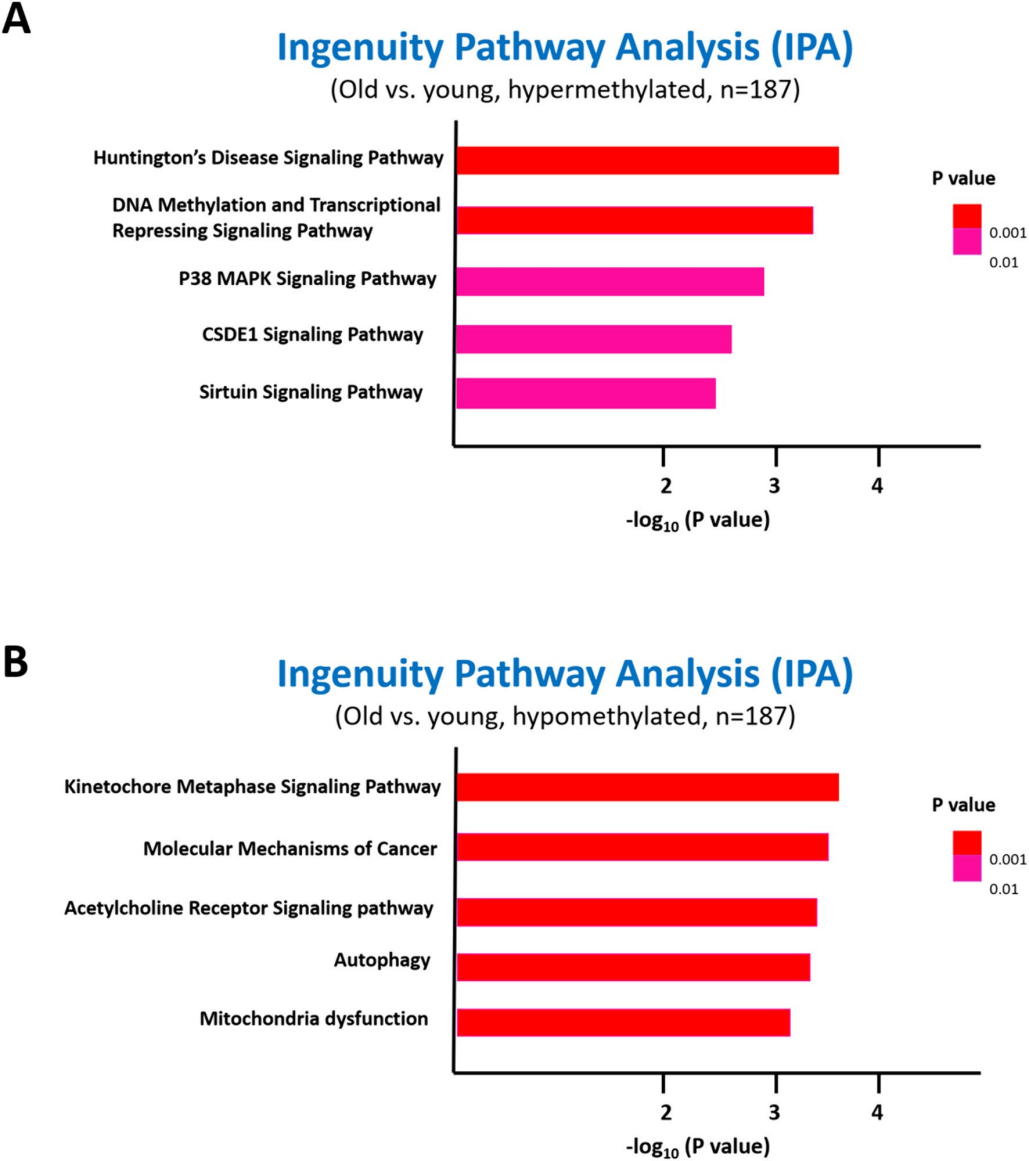
Ingenuity pathway analysis of **A** hypermethylated and **B** hypomethylated signaling pathways in comparison of old and young participants in subgroup with higher eGFR decline rate

Compared to younger people in subgroup of participants with higher eGFR decline rate (*n* = 187), hypermethylated genes in older participants involved in cellular process and regulation of gene expression and metabolism such as Huntington’s disease signaling pathway, DNA methylation and transcriptional repressing signaling pathway, P38 mitogen-activated protein kinase (MAPK) signaling pathway, cold shock domain-containing protein E1 (CSDE1) signaling pathway, and sirtuin signaling pathway (Fig. 3A). Conversely, several genes were hypomethylated in older participants which involved in basal cellular process and metabolism, including kinetochore metaphase signaling pathway, acetylcholine receptor signaling pathway, autophagy, and mitochondria dysfunction (Fig. 3B).

**Fig. 4:**
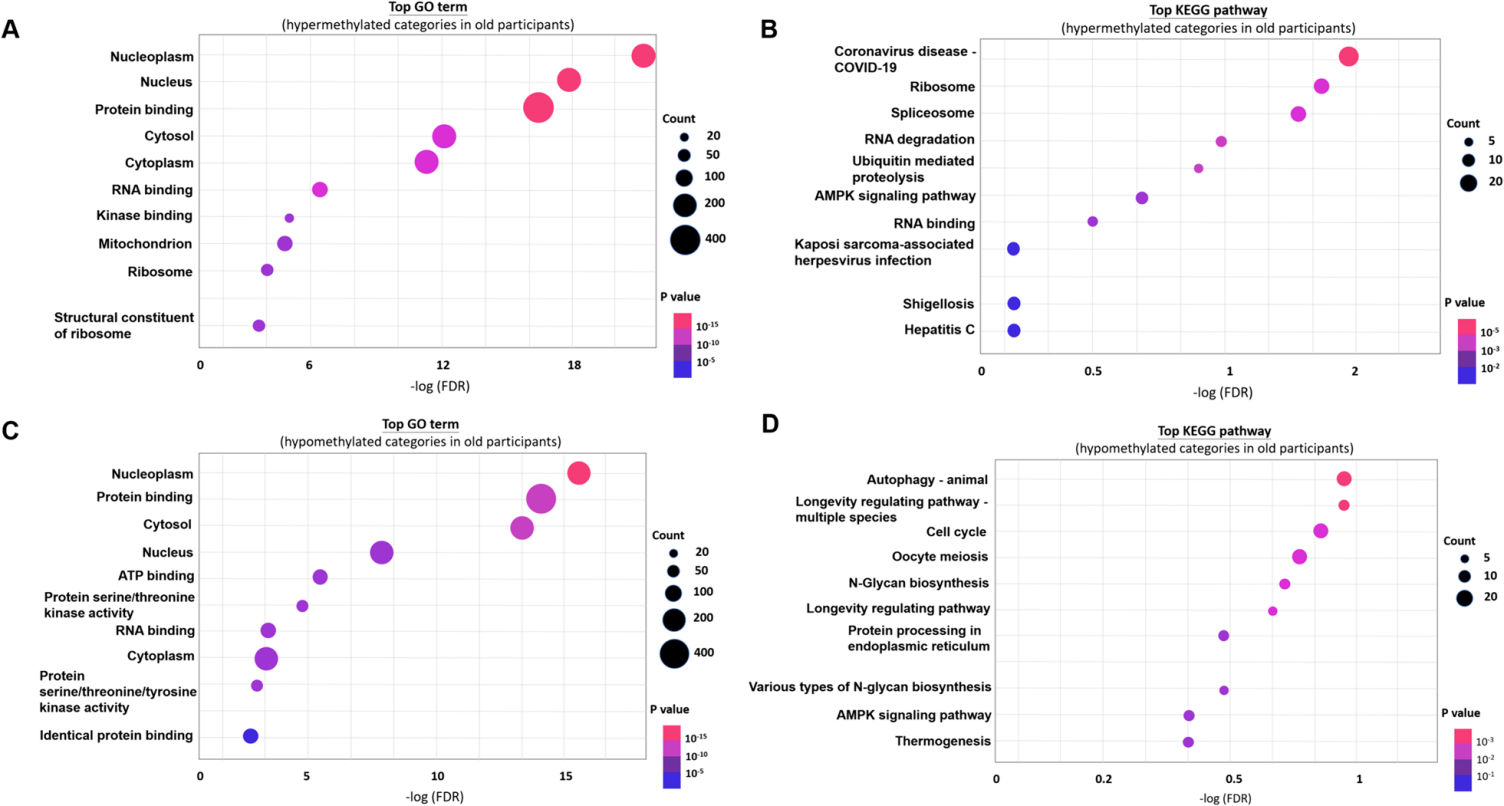
GO and KEGG pathway analysis in comparison of old and young participants in subgroup of participants with higher eGFR decline rate. **A**, **B** Top GO and KEGG pathway of hypermethylated genes. **C**, **D** Top GO and KEGG pathway of hypomethylated genes

In order to further analyze the enrichment of core genes both involved in renal function decline and aging, we conducted GO and KEGG pathway analysis in subgroup of participants with higher eGFR decline rate. By GO functional analysis, we identified that these hypermethylated genes in older participants were mainly related to cellular component (CC) and molecular function (MF) (Fig. 4A, Additional file 1: Table S1). Furthermore, KEGG pathway enrichment analysis showed that the hypermethylated genes in older participants were associated with the RNA degradation, ubiquitin mediated proteolysis, and AMP-activated protein kinase (AMPK) signaling pathways (Fig. 4B, Additional file 1: Table S2). On the other hand, hypomethylated genes in older participants were also associated with CC and MF (Fig. 4C, Additional file 1: Table S3) in GO analysis. KEGG pathway analysis demonstrated that these hypomethylated genes are associated with autophagy, longevity regulating pathway, and AMPK signaling pathway (Fig. 4D, Additional file 1: Table S4).

## Discussion

Our study investigated the association of DNA methylation in human PBMCs with aging and decline in renal function. Within the aging group and the group experiencing rapid eGFR decline, we identified several common hypermethylated genes, including *DNMT3A* and *GGACT*, as well as hypomethylated genes such as *ARL6IP5*, *CYB5D1*, *BCL6*, *RPRD2*, *ZNF451*, and *MIAT*. Additionally, we observed that the methylation status of several signaling pathways, particularly those related to autophagy and cytokine production, were associated with both aging and renal function decline. As the interplay between renal aging and renal disease is complex and the mechanisms of kidney disease may vary across different ethnicities, there has been limited research on the relationship between PBMCs' methylation status and aging as well as kidney diseases in Asian populations. Therefore, the genes and signaling pathways discovered in this study represent new directions for future research in this field.

Previous studies have demonstrated an association between the methylation status of PBMCs and kidney diseases, and it may be either causal or consequential. Analyzing the methylation status of PBMCs could uncover potential mechanisms for renal fibrosis and renal aging, or identify novel biomarkers with predictive power for renal outcomes. Using peripheral whole blood samples, an epigenome-wide association study (EWAS) was conducted, revealing that epigenetic changes in DNA methylation were associated with Type 1 diabetes and diabetic kidney disease [12, 13]. Moreover, Chu et al. employed EWAS to identify and validate CpGs, where methylation levels quantified from whole blood were associated with eGFR levels and the development of CKD in aging adults from population-based studies, including the ARIC Study and the Framingham Heart Study. They discovered 19 CpG sites significantly associated with eGFR/CKD, five of which were also associated with renal fibrosis in biopsies from CKD patients, demonstrating consistent DNA methylation changes in the kidney cortex [9]. Another study revealed associations between genome-wide DNA methylation patterns and both baseline eGFR levels and eGFR slopes of diabetic kidney disease. They displayed CpG sites located near genes such as *ZNF20* and *ITGB2*, which are known to be enriched for functional roles in kidney diseases. This underscored the potential of methylation markers in effectively stratifying the risk of kidney disease among individuals with type 2 diabetes [14]. As we know, DNA methylation promotes gene silencing and represents a common form of epigenetic modification [15]. The DNA methylation status, often referred to as the methylation clock, has emerged as a superior predictive biomarker for disease and mortality compared to chronological age [16]. This is particularly evident for genes involved in metabolic and DNA repair pathways, which exhibit predictive power across various tissue types and organisms [17]. All the aforementioned research demonstrates that investigating the methylation status of PBMCs as a mechanism or biomarker for studying kidney disease or renal aging is reasonable.

The hypermethylation of genes generally indicates a decrease in gene expression, further leading to a reduction in the activity of related signaling pathways. Hypomethylated genes, on the contrary, generally represent an increase in gene expression, consequently leading to an elevation in the activity of associated signaling pathways. In our study, autophagy-associated genes exhibited hypomethylation in participants with a higher eGFR decline rate compared to those with a lower eGFR decline rate. Furthermore, within the subgroup of participants with a higher eGFR decline rate, hypomethylation of autophagy-associated genes was also observed in older participants, strongly indicating an increase in autophagy activity. This suggests that autophagy activity in PBMCs may play a crucial role in both the decline of renal function and the aging process.

Autophagy is a cellular recycling process involving self-degradation and reconstruction of damaged organelles and proteins. Moreover, autophagy and cytokine interplay closely. Autophagy negatively regulates the processing and secretion of interleukin-1β (IL-1β) and IL-18, as well as the secretion of IL-1α, by macrophages and dendritic cells. Conversely, autophagy positively regulates the transcription and secretion of tumor necrosis factor-α (TNF-α), IL-8 and possibly IL-6. On the other hand, interferon (IFN)-γ, TNF-α, IL-1, IL-2, IL-6 and transforming growth factor-β (TGF-β) have been shown to induce autophagy, while IL-4, IL-10 and IL-13 are inhibitory [18]. In our study, hypomethylated autophagy-associated genes might indicate the increase of autophagy activity which might be induced by higher basal systemic inflammation of the older participants with higher eGFR decline rate. A previous study demonstrated that indoxyl sulfate increases the microtubule-associated protein 1A/1B-light chain 3 (LC3)-II/LC-I ratio in human T lymphocyte cells through N6-methyladenosine, suggesting that indoxyl sulfate may enhance autophagy flux [19]. Therefore, it is worth further investigating whether the autophagy activity of leukocytes induces renal disease and renal aging directly or indirectly damages the kidney by regulating inflammatory cytokines. Furthermore, if the alteration in autophagy activity of PBMCs reflects the aging process, this phenomenon could be similar in the renal aging process. Therefore, the autophagy activity of PBMCs could serve as a surrogate for renal aging and potentially predict the decline in renal function. There results shed light on its potential to develop the innovative diagnostic and therapeutic strategies.

The hypomethylated genes such as *ZNF451* and *ARL6IP5* identified in group with higher eGFR decline rate and older age are also correlated with autophagy. ZNF451 is involved in small ubiquitin-like modifier (SUMO)ylation, which is associated with the activation of autophagy. SUMOylation is a crucial process in various diseases, including pulmonary hypertension. In hypoxic pulmonary hypertension mouse models, a shift in the phenotype of pulmonary artery smooth muscle cells toward a synthetic phenotype was observed, accompanied by a notable increase in SUMO1 expression [20]. The mechanism underlying these effects is believed to involve the regulation of autophagy activation by SUMO1. SUMO1 induces the SUMOylation of vacuolar protein sorting 34 (Vps34) and subsequently, the formation of the autophagy initiation complex comprised of Beclin-1, Vps34, and autophagy-related gene 14-like protein [21, 22].

ADP-ribosylation factor-like 6 interacting protein 5 (ARL6IP5) binds and inhibits the cell membrane glutamate transporter, while also contributes to pleiotropic pathways [23]. ARL6IP5 is also a regulator of autophagy. In neurodegenerative disease, Siddique et al. demonstrated that ARL6IP5 can reduce the burden of α-synuclein aggregates and improve cell survival in a cellular model of Parkinson’s disease by inducing autophagy through the prevention of ubiquitination and degradation of autophagy-related 12 protein [24]. Nevertheless, ARL6IP5 can also suppress DNA repair and promote apoptosis pathways in ovarian carcinoma cells [25]. ARL6IP5 has been reported to be expressed in the kidney and is a potential causal candidate contributing to pleiotropic pathways between CKD and hyperuricemia [26]. Furthermore, the study of ARL6IP5 and kidney disease is limited, so the impact of ARL6IP5 on leukocytes and kidney disease warrants further investigation.

Our analysis also showed a positive correlation between the methylation status of the sirtuins (SIRTs) signaling pathway and age, suggesting a downregulation in the activity of the pathway during the aging process. SIRTs, which comprise a family of histone deacetylases with seven enzymatic activities in mammals (SIRT1–SIRT7), function to suppress gene transcription through epigenetic mechanisms. Nuclear sirtuins, notably SIRT 1, 2, 6, and 7, may play a significant role in regulating inflammatory responses [27]. Moreover, studies have demonstrated that overexpression of SIRT1 results in reduced nuclear factor kappa-light-chain-enhancer of activated B cells (NF-κB) activity [28], while knockdown of SIRT1 in macrophages increases lipopolysaccharides-stimulated TNF-α secretion [29]. Other research has indicated that SIRT1-mediated deacetylation of NF-κB inhibits inducible nitric oxide synthase and cytokine-mediated beta-cell damage in isolated rat islets [30]. Additionally, investigations showed that SIRT1 also deacetylates and suppresses the transcription activity of activator protein-1, leading to a downregulation of cyclooxygenase-2 gene expression [31, 32]. These findings suggest the presence of multiple targets of SIRT1 with potential roles in the downregulation of inflammation. Hence, our study uncovered that hypomethylation of genes related to the sirtuin signaling pathway in the aging and higher eGFR decline group could potentially elevate inflammation levels, leading to renal aging and kidney damage.

Furthermore, we observed hypermethylation of the gene associated with p38 MAPK signaling pathway, particularly mitogen- and stress-activated protein kinase 1(MSK1). The p38 MAPK signaling pathway regulates cytokine production, including IL-1β, TNF-α, and IL-10 [33]. Two downstream kinases of p38 are mitogen-activated protein kinase-activated protein kinase 2 (MK2) and (MSK1/2). MK2 can increase TNF-α, IL-1β, and IL-6 levels, leading to a pro-inflammatory response that can further activate the same pathway and amplify inflammation [34]. MSK1/2, on the other hand, deactivates p38 and contributes the suppression loop of p38, which includes two anti-inflammatory cytokines, IL-1 receptor antagonist and IL-10. These cytokines have the potential to counteract the effects of IL-1β and suppress the inflammatory response [35]. Therefore, hypermethylation of MSK1 might decrease its gene expression, disrupting the suppression loop of the p38 MAPK signaling pathway, and contributing to inflammation-induced renal aging and kidney disease.

There are several limitations in this study. Firstly, we were unable to directly confirm the consistency of methylation status between PBMCs and renal tissue and thus could not analyze the correlation between PBMC methylation status and the severity of renal disease directly. Secondly, the follow-up period was relatively short, probably reducing the accuracy of assessing the impact of PBMC methylation status on the kidneys over time. Thirdly, not all patients had urine protein data available, which could limit the evaluation of kidney function, as proteinuria is a crucial marker of kidney disease. Additionally, out of 143,070 participants, only 1,587 agreed to blood sampling for whole blood genome-wide methylation analysis and follow-up serum creatinine measurements. We could not avoid potential selection bias, which may affect the results. Finally, other potential confounders, such as sex or coexisting conditions, may affect the results. Future research is needed to investigate the impact of these specific differences on DNA methylation. Nevertheless, the results obtained from this study are still valuable due to the precious data on PBMC methylation status in the Asian population.

## Conclusion

In summary, through methylation analysis of PBMCs, we have identified significant genes such as *ZNF451* and *ARL6IP5*, as well as signaling pathways such as autophagy, p38 MAPK, and sirtuin, which might play crucial roles in aging and the decline of renal function. These findings contribute to the development of novel biomarkers for identifying at-risk groups and even for therapeutic agent discovery. Unlike genetic changes, epigenetic alterations are potentially reversible, offering opportunities for therapeutic interventions. Further prospective studies are needed to determine whether the observed methylation influences are causal or consequential. The identification of unique epigenetic profiles associated with aging and higher eGFR decline could shed light on additional biological mechanisms underlying renal aging and kidney disease.

## Methods

### Data source and study population

We utilized the Taiwan Biobank, a prospective cohort study that enrolls general population of Taiwanese residents aged 30 to 70 years with no gender restrictions. Individuals of foreign nationality, foreign ancestry, and/or those diagnosed with cancer were excluded. The decision to perform whole blood genome-wide methylation analysis and follow-up serum creatinine measurements was based on their informed consent. Apart from this, there were no other exclusion criteria. This study involved 143,070 participants who were surveyed via questionnaires, underwent physical examinations, and provided biological samples between January 1, 2012, and December 31, 2021 [36]. We specifically included participants with both whole blood genome-wide methylation data and follow-up data on serum creatinine. The average interval between the first and the second eGFR measurements was 4.19 years. And we used CKD-EPI formula to calculated eGFR [37].

The participants were divided into rapid and non-rapid eGFR decline groups based on a cutoff value of 3.3% per year of eGFR decline. The first reason for choosing this threshold was that the population in our study was from the community, with an average baseline eGFR of 101.54 ml/min/1.73m^2^, which was much higher than that of CKD patients. Therefore, the proportion of decline in eGFR may be more appropriate to consider than the absolute level of eGFR decline. Secondly, previous studies characterized progressive renal decline as an eGFR reduction of ≧ 3.3% per year [38, 39]. In a study of the general population, this threshold represented the 2.5th percentile of the annual renal function reduction distribution [40]. As a result, we chose 3.3% per year as the cutoff for distinguishing between rapid and non-rapid eGFR decline groups to identify more significant results in the following analysis. Figure 1 displays a flow diagram of the study.

### DNA methylation array analysis

The Illumina MethylationEPIC array (Illumina, Inc.) was employed to assess the genome-wide methylation. The array designed with the CpG annotation was obtained through the IlluminaHumanMethylationEPICmanifest package based on the Infinium MethylationEPIC v1.0 manifest file. The minfi package for analyzing Illumina's Methylation arrays was utilized to process raw image data chunk (IDAT) files of the dataset for R software. The detection P values were calculated for each probe by comparing the total signal (methylated + unmethylated signal) to the background. A mean detection *P* value of > 0.05 was used as a cutoff for removing poor-quality samples. To reduce the technical variation within and between samples, quantile normalization was implemented for the dataset. Poor-performing probes were excluded, which failed in samples with poor average detection *P* (> 0.01). The removal of probes included those affected by common single-nucleotide polymorphisms (SNPs) or showed to be cross-reactive. The remaining 754,325 probes were adopted for downstream analysis. Differentially methylated probes (DMPs) were analyzed, and their associated genes were identified between higher and lower eGFR decline group (overall cohort, *n* = 1587). Differentially methylated regions (DMRs) were performed using the DMRcate package. DMRs and their corresponding genes were identified based on the DMPs with an adjusted *P* value (Benjamini–Hochberg) of < 0.05. The signaling pathways associated with these genes were analyzed using QIAGEN ingenuity pathway analysis (IPA) (Additional file 1: Fig. S1).

In analysis of subgroup with higher eGFR decline (decline rate > 3.3%/year, *n* = 187), participants were grouped according to age (age < 45 years as group 1, 45 years ≤ age < 65 years as group 2, and age ≥ 65 years as group 3). DMPs across groups (group 3—group 2, and group 2—group 1) were identified by the linear regression model using the limma package. Only DMRs, in which DMP methylated levels increase with age (fold change > 0 in both group 3—group 2 and group 2—group 1) or decrease with age (fold change < 0 in both group 3—group 2 and group 2—group 1), were adopted for the gene ontology analysis. Gene ontology analysis on those DMRs was performed using the missMethyl package. Kyoto Encyclopedia of Genes and Genomes (KEGG) pathways, the Gene Ontology (GO) and C2 curated genesets from the Broad Institute Molecular signatures database (MSigDB) were employed to perform enrichment analysis. Only top unique terms were shown. Additionally, these DMRs and their corresponding genes were analyzed using QIAGEN ingenuity pathway analysis (IPA).

### Statistical analyses

SAS software version 9.4 (SAS Institute Inc., Cary, NC, USA.) was used for statistical analyses. Continuous variables are presented as the mean and standard deviation, and categorical variables are summarized as percentages. The comparison between participants with higher or lower eGFR decline rates was made using a two-tailed Student’s *t* test. A *P* value less than 0.05 was considered statistically significant.

### Supplementary Information

**Additional file 1: Fig. S1**. Workflow of methylation analysis. **Fig. S2**. Ingenuity Pathway Analysis (IPA) of (A) hypermethylated signaling pathways and (B) hypomethylated signaling pathways in participants with a higher eGFR decline rate

## Data Availability

The data of this article cannot be shared publicly due to the privacy concerns of individuals who participated in the study. However, the data will be made available upon reasonable request to the corresponding author.
