# Supplementary material for: DNA methylation in peripheral blood is associated with renal aging and renal function decline: a national community study
Source: Clin Epigenetics. 2024 Jun 15;16:80. doi: 10.1186/s13148-024-01694-y (PMC11180394; doi:10.1186/s13148-024-01694-y)

**DNA methylation in peripheral blood is associated with renal aging and renal function decline: a national community study**

Po-Lung Yang^1^, Tai-Shuan Lai^2,3^, Yu-Hsiang Chou^2,*^, Liang-Chuan Lai^4,5^, Shuei-Liong Lin^2,4,6^, Yung-Ming Chen^2,7^

**Authors’ Affiliations and addresses:** ^1^Department of Geriatrics and Gerontology, National Taiwan University Hospital, College of Medicine, National Taiwan University, Taipei, Taiwan; ^2^Renal Division, Department of Internal Medicine, National Taiwan University Hospital, College of Medicine, National Taiwan University, Taipei, Taiwan; ^3^Institute of Epidemiology and Preventive Medicine, College of Public Health, National Taiwan University, Taipei, Taiwan; ^4^Graduate Institute of Physiology, College of Medicine, National Taiwan University, Taipei, Taiwan; ^5^Bioinformatics and Biostatistics Core, Center of Genomic and Precision Medicine, National Taiwan University, Taipei, Taiwan; ^6^Research Center for Developmental Biology and Regenerative Medicine, National Taiwan University, Taipei, Taiwan; ^7^Department of Internal Medicine, National Taiwan University Hospital, Bei-Hu branch, Taipei, Taiwan

**Supplementary Table S1.** **Top 10 GO terms of hypermethylated genes in older participants with a higher eGFR decline rate**

| **Functional**  **Category** | **Term** | **Genes** | **Fold enrichment** | **FDR** |
| --- | --- | --- | --- | --- |
| CC | GO:0005654~nucleoplasm | TCERG1, IPO11, ZMYND8, FCF1, IKZF1, CREB3L4, MYC, OGFOD1, SPATA24, EPC1, TRIM27, BTRC, ANKS1A, TIPIN, SMARCC1, METTL3, MRPS18B, WDR74, DNTTIP2, PRKCA, ARMC8, ZNF12, PRKAB1, RRAGA, DDX39B, SUB1, XRN2, TRIM14, ASF1B, PARPBP, DHX8, NEDD4L, NLK, ANAPC10, ZBTB | 1.939 | 1.395E-21 |
| CC | GO:0005634~nucleus | TCERG1, GABPB2, RPL3, CCNI, ZMYND8, LRR1, IKZF1, NUDT5, NUDT6, RPL9, RPL7, ZSCAN30, CREB3L4, MYC | 1.629 | 4.158E-18 |
| MF | GO:0005515~protein binding | IPO11, GABPB2, RPL3, WIPF1, RAPH1, RPL9, GLS, RPL7, ZSCAN30, CREB3L4, MYC, WDR90, LUC7L, RPL38, ANKS1A | 1.237 | 1.697E-14 |
| CC | GO:0005829~cytosol | IPO11, RPL3, WIPF1, LRR1, RAPH1, IKZF1, NUDT5, RPL9, GLS, RPL7, HERC5, HERC3, RASSF3, OGFOD1, SPATA24, RPL38, RPL37, TRIM27, BTRC, ANKS1A, SEPHS2, METTL3, COG3, ALG2, DLEC1, ANK2, PRKCA, ARMC8, FNDC3A, PRKAB1, GNPAT, TIAM1, RRAGA, TRIM14, VPS28, ASF1B, TM | 1.591 | 4.097E-14 |
| CC | GO:0005737~cytoplasm | IPO11, RPL3, CCNI, ZMYND8, NUDT6, RPL9, RPL7, HERC5, HERC3, RASSF3, WDR90, OGFOD1, SPATA24, EPC1 | 1.551 | 1.021E-12 |
| MF | GO:0003723~RNA binding | TCERG1, RBM25, DAZAP1, PUS10, RPL3, HSP90AB1, CELF1, FCF1, SRP54, HSPB1, PARK7, KRI1, AATF, RPL9, RPL7 | 2.104 | 1.025E-08 |
| MF | GO:0019900~kinase binding | HSP90AB1, CAB39, DAB2IP, TNFAIP3, PARK7, EGFR, MYOM2, JAKMIP3, MOB4, DUSP12, TIAM1, LDHB, RPS3, PFKM | 5.390 | 6.130E-05 |
| CC | GO:0005739~mitochondrion | CREBZF, PUS10, CLIC4, MTCH2, ALAS1, HSP90AB1, OSGEPL1, CISD1, MRPL36, PARK7, NUDT6, MYOM2, GLS | 1.813 | 1.506E-04 |
| CC | GO:0005840~ribosome | GADD45GIP1, MRPS28, RPL3, MRPS36, MRPS18B, RPL35A, RPS27L, RPL9, MRPL3, RPS27, OAS1, TBCE, RPS3, RPL14 | 3.919 | 3.252E-04 |
| MF | GO:0003735~structural constituent of ribosome | RPL3, MRPS18B, RPL35A, RPS27L, MRPL36, RPL9, RPL7, MRPL52, MRPL3, MRPL1, RPS27, RPL7L1, TBCE, RPS3 | 3.652 | 4.718E-04 |

GO = Gene Ontology; FDR = false discovery rate; CC = cellular component; MF = molecular function

**Supplementary Table S2. Top 10 KEGG pathway of hypermethylated genes in older participants with a higher eGFR decline rate**

| **Term** | **Genes** | **Fold enrichment** | **FDR** | **P value** |
| --- | --- | --- | --- | --- |
| hsa05171: Coronavirus disease - COVID-19 | RPL3, STAT2, RPL35A, RPS27L, PRKCA, TYK2, RPL9, EGFR, RPL7, TNFRSF1A, RPS27, OAS1, RPS3, RPL14, RPL38 | 2.785 | 0.027 | 9.636E-05 |
| hsa03010: Ribosome | RPL3, RPL35A, RPS27L, MRPL36, RPL9, RPL7, MRPL3, MRPL1, RPS27, RPS3, RPL14, RPL38, RPL37, RPS27A, RSL24D1, RPL19 | 3.095 | 0.027 | 1.913E-04 |
| hsa03040: Spliceosome | TCERG1, PRPF38B, RBM25, DHX8, PRPF40A, LSM5, LSM3, LSM6, HNRNPK, DDX39B, RNU5B-1, SRSF4, HNRNPC | 2.531 | 0.104 | 0.001 |
| hsa03018: RNA degradation | CNOT6, LSM6, PNPT1, XRN2, C1D, LSM5, DCP1A, PFKM, LSM3 | 3.681 | 0.207 | 0.002 |
| hsa04120: Ubiquitin mediated proteolysis | PIAS4, DDB1, UBE2W, HERC3, ANAPC7, UBA2, NEDD4L, BTRC, CBL, RPS27A, ANAPC10, ANAPC1 | 2.730 | 0.251 | 0.004 |
| hsa04152: AMPK signaling pathway | RPTOR, TBC1D1, CAB39, CREB3L4, RPS6KB2, PPP2R5C, PRKAB1, PFKM, FOXO1, RAB11B | 2.670 | 0.590 | 0.012 |
| hsa05167: Kaposi sarcoma-associated herpesvirus infection | CREBBP, STAT2, NFATC1, TYK2, TNFRSF1A, CASP9, HCK, CASP8, MAP1LC3A, GNG5, MYC, RPS27A, JAK1 | 2.165 | 0.668 | 0.017 |
| hsa05131: Shigellosis | FOXO1, EGFR, TNFRSF1A, RPTOR, DIAPH1, RRAGA, MAP1LC3A, RPS6KB2, HCLS1, ATM, BTRC, RPS27A, PPID, CYTH1, BCAR1 | 1.962 | 0.668 | 0.021 |
| hsa05160: Hepatitis C | CASP9, CASP8, RSAD2, OAS1, MYC, STAT2, TYK2, LDLR, EGFR, JAK1, TNFRSF1A | 2.263 | 0.668 | 0.023 |
| hsa04137: Mitophagy - animal | FIS1, MAP1LC3A, RRAS, CITED2, SP1, MITF, RPS27A | 3.141 | 0.668 | 0.023 |

KEGG = Kyoto Encyclopedia of Genes and Genomes; FDR = false discovery rate

**Supplementary Table S3. Top 10 GO terms of hypomethylated genes in older participants with a higher eGFR decline rate**

| **Functional**  **Category** | **Term** | **Genes** | **Fold enrichment** | **FDR** |
| --- | --- | --- | --- | --- |
| CC | GO:0005654~nucleoplasm | SCOC, DCAF8, SMC3, DCAF6, PRKACG, ANKS1A, DDX17, SP110, RUNX2, RUNX1, THAP4, EID2, NEIL3, PSME3 | 1.817 | 2.614E-16 |
| MF | GO:0005515~protein binding | SCOC, GABPB2, RPL3, ZFYVE9, DCAF8, SMC3, DCAF6, CAPNS1, PRKACG, WDR90, GPBP1, ANKS1A, DDX17, ARMC9 | 1.227 | 1.436E-12 |
| CC | GO:0005829~cytosol | SCOC, RPL3, ZFYVE9, DCAF8, NADSYN1, NUDT5, SMC3, DCAF6, CAPNS1, RPL18A, FTH1, PRKACG, GPBP1, MLYCD | 1.550 | 6.121E-12 |
| CC | GO:0005634~nucleus | GABPB2, RPL3, GFI1, CPNE7, DCAF8, NUDT5, SMC3, DCAF6, FTH1, PRKACG, GPBP1, CCNL1, DDX17, SP110, RFX2 | 1.406 | 2.938E-07 |
| MF | GO:0005524~ATP binding | TOP2A, GSK3A, NVL, MCM9, PARS2, NAT10, BUB1B, PRKAG2, DDX60L, NADSYN1, SMC3, CHD2, PFAS, IGF1R | 1.947 | 1.385E-06 |
| MF | GO:0004674~protein serine/threonine kinase activity | CAMK2D, GSK3A, PRKAA1, PRKDC, PIK3R4, BUB1B, ACVR1B, PPM1D, PRKCZ, AURKA, NRBP2, MAPK7, GRK5, PRKACG, STK38, PHKG2, NEK4, CSNK1A1, VRK1, EIF2AK4, VRK3, CIT, CLK1, OBSCN, WNK1, CDK4, WNK2, AAK1, ULK3, CDK1, ATM, SQSTM1 | 2.842 | 9.614E-05 |
| MF | GO:0003723~RNA binding | TOP2A, RPL3, PPP1R10, NVL, CCDC47, NAT10, C1ORF131, DDX60L, MSI2, CHD2, CASP7, SART3, RPL18A, SIN3A | 1.782 | 2.797E-04 |
| CC | GO:0005737~cytoplasm | RPL3, UXS1, CPNE7, DCAF8, DDX60L, NADSYN1, DCAF6, CYB5D1, CAPNS1, RPL18A, WDR90, FTH1, MLYCD | 1.327 | 3.428E-04 |
| MF | GO:0004712~protein serine/threonine/tyrosine kinase activity | CAMK2D, GSK3A, PRKAA1, PRKDC, PIK3R4, BUB1B, PRKCZ, AURKA, IGF1R, MAPK7, PRKACG, STK38, FYN, JAK3 | 2.490 | 0.001 |
| MF | GO:0042802~identical protein binding | CREBZF, GABPB2, GMEB2, CSF1, UXS1, PARS2, AMD1, MSI2, NUDT5, LITAF, IGF1R, PCM1, FTH1, TRIM3, ANXA6, GRB10, MLYCD, CCHCR1, EIF2B1, DNM1L, PRMT5, PCYT1A, DYNLT1, GGPS1, EED, COG4, LZTFL1, TSN, FOXP1, THAP4, IRF3, SOAT1, CLDN9, PSME3, TIMELESS, VWA1, MAPRE3 | 1.597 | 0.005 |

GO = Gene Ontology; FDR = false discovery rate; CC = cellular component; MF = molecular function; ATP = Adenosine triphosphate

**Supplementary Table S4. Top 10 KEGG pathway of hypomethylated genes in older participants with a higher eGFR decline rate**

| **Term** | **Genes** | **Fold enrichment** | **FDR** | **P value** |
| --- | --- | --- | --- | --- |
| hsa04140: Autophagy - animal | RAB1A, PRKAA1, PIK3R4, ITPR1, CFLAR, AMBRA1, EIF2AK4, IGF1R, RAB33B, RPTOR, PRKACG, AKT1S1, BCL2 | 3.045 | 0.118 | 6.498E-04 |
| hsa04213: Longevity regulating pathway - multiple species | RPTOR, HSPA8, PRKAA1, PRKACG, INSR, AKT1S1, PRKAG2, ADCY2, IGF1R | 4.452 | 0.118 | 8.263E-04 |
| hsa04110: Cell cycle | ZBTB17, ANAPC7, PRKDC, BUB1B, DBF4B, PPP2R5C, SMC3, FZR1, ORC1, CDK4, ORC3, CDK1, ANAPC5, ATM | 2.735 | 0.167 | 0.002 |
| hsa04114: Oocyte meiosis | CAMK2D, ANAPC7, PRKACG, CDK1, ITPR1, ANAPC5, ADCY2, PPP2R5C, SMC3, CALM2, AURKA, IGF1R | 2.809 | 0.250 | 0.004 |
| hsa00510: N-Glycan biosynthesis | GANAB, OSTC, ALG14, ALG3, MAN1A1, MGAT2, STT3B | 4.051 | 0.389 | 0.007 |
| hsa04211: Longevity regulating pathway | RPTOR, PRKAA1, PRKACG, INSR, AKT1S1, PRKAG2, ADCY2, CREB5, IGF1R | 3.101 | 0.389 | 0.008 |
| hsa04141: Protein processing in endoplasmic reticulum | HSPA8, VCP, EDEM2, EIF2AK4, RAD23B, HSP90B1, GANAB, BAG2, OSTC, BCL2, MAN1A1, STT3B, UGGT1 | 2.291 | 0.398 | 0.011 |
| hsa00513: Various types of N-glycan biosynthesis | OSTC, ALG14, ALG3, MAN1A1, MGAT2, STT3B | 4.381 | 0.398 | 0.011 |
| hsa04152: AMPK signaling pathway | RPTOR, PRKAA1, INSR, AKT1S1, PRKAG2, PPP2R5C, MLYCD, CREB5, IGF1R, RAB11B | 2.535 | 0.478 | 0.017 |
| hsa04714: Thermogenesis | SMARCD1, PRKAA1, COX15, NDUFA4, COX17, PRKAG2, FRS2, ADCY2, RPTOR, CPT2, PRKACG, AKT1S1, NDUFV2 | 1.983 | 0.478 | 0.019 |

KEGG = Kyoto Encyclopedia of Genes and Genomes; FDR = false discovery rate; AMPK = AMP-activated protein kinase

**Supplementary Fig. S1. Workflow of methylation analysis**


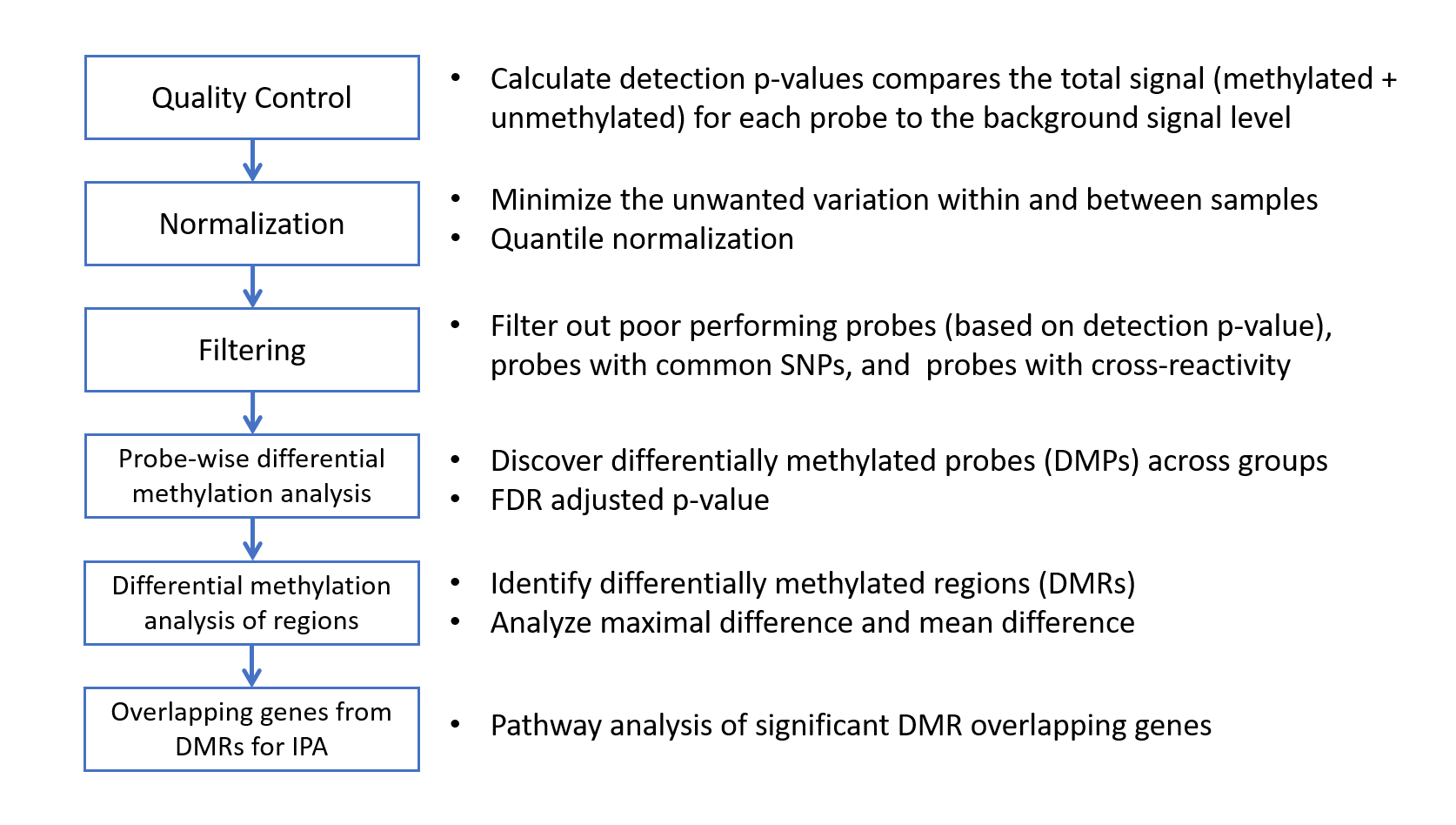


SNP = single-nucleotide polymorphism; FDR = false discovery rate

**Supplementary Fig. S2. Ingenuity Pathway Analysis (IPA) of (A) hypermethylated signaling pathways and (B) hypomethylated signaling pathways in participants with a higher eGFR decline rate**


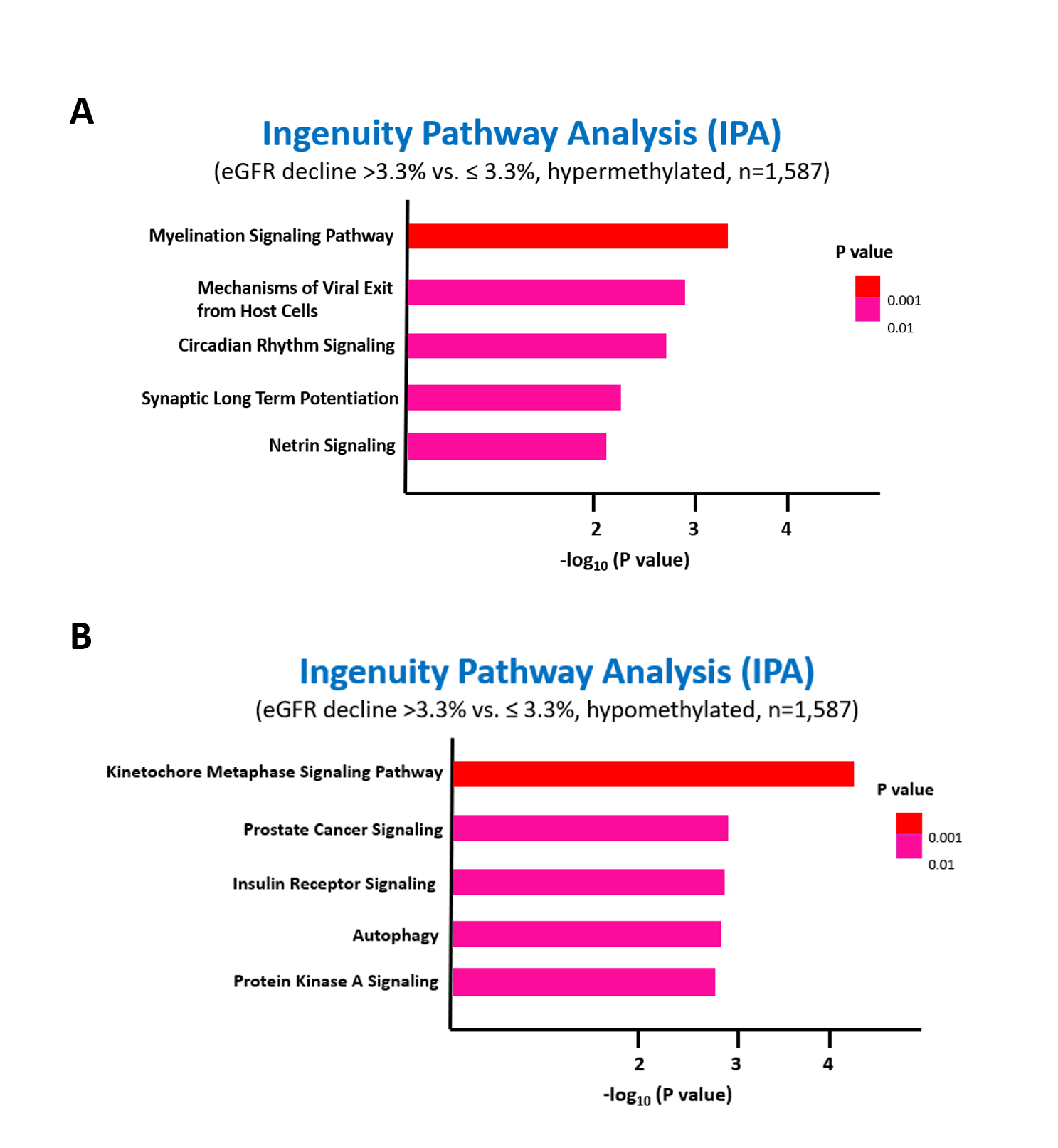

Supplement: Supplementary file 1 — Additional file 1: Fig. S1. Workflow of methylation analysis. Fig. S2. Ingenuity Pathway Analysis (IPA) of (A) hypermethylated signaling pathways and (B) hypomethylated signaling pathways in participants with a higher eGFR decline rate [file 13148_2024_1694_MOESM1_ESM.docx]
